# Can the adverse childhood experiences (ACEs) checklist be utilized to predict emergency department visits among children and adolescents?

**DOI:** 10.1186/s12874-021-01392-w

**Published:** 2021-09-25

**Authors:** Asmita Bhattarai, Gina Dimitropoulos, Brian Marriott, Jaime Paget, Andrew G. M. Bulloch, Suzanne C. Tough, Scott B. Patten

**Affiliations:** 1 Department of Community Health Sciences, Cumming School of Medicine, University of Calgary, 3280 Hospital Drive NW, Calgary, AB T2N4Z6 Canada; 2 Mathison Centre for Research & Education, University of Calgary, 3280 Hospital Drive NW, Calgary, AB T2N4Z6 Canada; 3 Faculty of Social Work, University of Calgary, 2500 University Dr NW, Calgary, AB T2N 1N4 Canada; 4 Addiction and Mental Health, Alberta Health Services- Calgary Zone, Calgary, AB Canada; 5 Department of Psychiatry, Cumming School of Medicine, University of Calgary, 2500 University Dr NW, Calgary, AB T2N 1N4 Canada; 6 Department of Pediatrics, Cumming School of Medicine, University of Calgary, 2500 University Dr NW, Calgary, AB T2N 1N4 Canada

**Keywords:** ACEs, Adverse childhood experiences, Emergency department visit, Prediction, Least absolute shrinkage and selection operator, Mental illness

## Abstract

**Background:**

Extensive literature has shown an association of Adverse Childhood Experiences (ACEs) with adverse health outcomes; however, its ability to predict events or stratify risks is less known. Individuals with mental illness and ACE exposure have been shown to visit emergency departments (ED) more often than those in the general population. This study thus examined the ability of the ACEs checklist to predict ED visits within the subsequent year among children and adolescents presenting to mental health clinics with pre-existing mental health issues.

**Methods:**

The study analyzed linked data (*n* = 6100) from two databases provided by Alberta Health Services (AHS). The Regional Access and Intake System (RAIS 2016–2018) database provided data on the predictors (ACE items, age, sex, residence, mental health program type, and primary diagnosis) regarding children and adolescents (aged 0–17 years) accessing addiction and mental health services within Calgary Zone, and the National Ambulatory Care Reporting System (NACRS 2016–2019) database provided data on ED visits. A 25% random sample of the data was reserved for validation purposes. Two Least Absolute Shrinkage and Selection Operator (LASSO) logistic regression models, each employing a different method to tune the shrinkage parameter lambda (namely cross-validated and adaptive) and performing 10-fold cross-validation for a set of 100 lambdas in each model were examined.

**Results:**

The adaptive LASSO model had a slightly better fit in the validation dataset than the cross-validated model; however, it still demonstrated poor discrimination (AUC 0.60, sensitivity 37.8%, PPV 49.6%) and poor calibration (over-triaged in low-risk and under-triaged in high-risk subgroups). The model’s poor performance was evident from an out-of-sample deviance ratio of − 0.044.

**Conclusion:**

The ACEs checklist did not perform well in predicting ED visits among children and adolescents with existing mental health concerns. The diverse causes of ED visits may have hindered accurate predictions, requiring more advanced statistical procedures. Future studies exploring other machine learning approaches and including a more extensive set of childhood adversities and other important predictors may produce better predictions. Furthermore, despite highly significant associations being observed, ACEs may not be deterministic in predicting health-related events at the individual level, such as general ED use.

**Supplementary Information:**

The online version contains supplementary material available at 10.1186/s12874-021-01392-w.

## Background

Adverse childhood experiences (ACEs) refer to a set of stressors during childhood [1, 2]. The first ACE study measured ten types of childhood adversities experienced by an individual before 18 years of age, namely physical abuse, emotional abuse, sexual abuse, emotional neglect, physical neglect, witnessing interpersonal violence, parental divorce, parental substance abuse, living with a family member with mental illness, and incarceration of a family member [1]. The ACEs have been used as a checklist or a tool to measure exposure to childhood adversities by subsequent studies [3–5] and might also have usefulness in clinical application in the context of therapeutic dialogue and trauma-informed care [6–9].

With various studies reporting the prevalence of at least one ACE to be over 50% [1, 10], ACEs are commonplace. These adversities are associated with poor health and well-being throughout life, the adverse outcomes ranging from smoking, alcoholism, drug abuse, suicide attempts, chronic diseases, mental illness, and even mortality [1, 3–5, 11]. Most of these ACE-associated adverse health conditions are common reasons for people to access acute health care services [12], notably, increased uptake of emergency department (ED) services [11, 13–17]. For instance, one Canadian study reported that, compared to the general population, people with more than one ACE, visit the emergency department up to 29% more often than those without the exposure [10]. Another study among children and adolescents reported that those who reported childhood abuse had a higher number of ED visits than their counterparts (2.1 vs. 1.5, *p*-value < 0.01) [18]. Individuals with mental illness are also more likely to visit the ED more frequently than those without mental illness [11]. Studies have reported that as many as 25–30% of the ED visitors have been noted to have a mental illness [19, 20]. Both ACE exposure and mental illnesses can contribute to overcrowding in emergency departments and place administrative and economic burdens on the health system [11, 19–21].

Preventing ED visits may be a means of reducing medical costs [13, 17, 22, 23], which is the inherent interest of health care reformers and policymakers since those resources could be directed elsewhere to achieve more efficient and equitable service delivery [24]. It has been reported that youth with childhood maltreatment have higher health care utilization costs, and increased ED visits contribute in part to this higher overall cost [25]. Accessing acute health care may indicate positive health-seeking behavior; however, if it is recurrent and is preventable with primary care, it may reflect ineffectiveness in care pathways; especially in educating patients regarding the importance of accessing primary care, assisting them with scheduling appointments, and following them up to monitor them adequately [13, 17, 26, 27].

ACE data has been used in public health programs for various health promotion and illness prevention efforts [28] and is being routinely collected in various health service programs across Canada. However, clinicians and researchers question whether it can be used to predict salient health care events such as future ED visits at the individual level. Being statistically associated with health care outcomes is not the same as being predictive of those outcomes. A significant association of ACEs with ED does not confirm that outcomes can be accurately predicted [29]. An ability to predict this outcome at the individual level could help implement targeted interventions to improve clinical care and reduce emergency visits and unplanned readmissions [13, 14, 17, 30]. If childhood adversities are as strongly associated with adverse health outcomes such as suicidal behavior, sexually transmitted infections, problematic substance use in youth and adults as reported by studies [1–3], then they may be useful to predict ED visits. Then, the health care providers would be able to utilize predictions to improve outpatient health services’ effectiveness and reduce the burden to emergency and inpatient services [10, 13]. The health service utilization data collected in the Canadian health care system, known as administrative databases (since the data are used in the administration of health services), provides us the opportunity to link the ACE data with prospective follow-up of ED visits data and to evaluate the predictive ability of ACE items, combined with other routinely collected data elements, in predicting the risk of ED visits [31]. The results may contribute to advance the field towards targeted preventive care and, in turn, reduce health care costs.

Machine learning approaches are gaining more attention as a tool for prediction in research and clinical practice. Many studies have reported that machine learning can better predict patient outcomes (provide more stable predictions) than traditional modeling approaches based on non-regularized regression in various disease conditions and settings [32–35]. These algorithms are even more applicable in health care data since they can account for the interactions, unusual distributions, and a large number of variables [34, 36]. Researchers and clinicians interested in using ACEs data to predict health outcomes have mostly relied on traditional modeling approaches and focused on psychopathological and behavioral outcomes. There is not much evidence around the predictive ability of childhood adversities for important health-related events such as ED visits, irrespective of the prediction modeling approaches (i.e., traditional and machine learning). Studies examining the characteristics of frequent ED users have also failed to examine childhood adversities as potential risk factors for increased ED visits [37]. Hence, it is worth exploring whether a machine learning approach can lead to a prediction that is accurate enough to support clinical decisions and build strategies to intervene in advance to avoid future adverse health events such as ED visits [32, 38, 39]. The least absolute shrinkage and selection operator (LASSO) models are gaining popularity in prediction since they have better interpretability than other less transparent machine learning approaches [35, 40]. The models are developed in a training dataset followed by testing or validation in a new dataset. Also, through regularization (shrinkage), LASSO is believed to reduce overfitting while dealing with many predictors [40], leading to strong prediction performance.

This study aims to address the existing knowledge gap and provide clinicians a potential resource to guide their clinical decision-making by using the routinely collected and readily available patient information. The study thus examined the ability of specific childhood adversities, measured using the ACE checklist, in predicting the risk of ED visits within the subsequent year among children and adolescents with mental illness presenting to outpatient addiction and mental health services program. We employed LASSO as a machine learning modeling approach.

### Objective

The objective of the study was to evaluate the predictive performance of the machine learning model incorporating ACEs and routinely available variables to predict subsequent one-year ED visits among children and adolescents seen for pre-existing addiction or mental health concerns in an outpatient setting.

## Methods

### Data source

The data was available through record linkage of two sizable databases, namely, Regional Access and Intake System (RAIS; a local health service registration database) and National Ambulatory Care Repository System (NACRS; a national health administrative database). The RAIS database, developed as a client tracking system to support the Alberta Health Services’ (AHS) Child and Adolescent Addiction and Mental Health and Psychiatry Program (CAAMHPP), provided information on multiple predictors [41, 42]. The CAAMHPP is a regional program serving a geographically defined catchment area, i.e., Calgary Zone in Alberta, and consists of several services, including inpatient, outreach, community and outpatient, day programs, school-based services, and specialized services. These services are typically provided for more complex/severe clients requiring a higher level of addiction or mental health service versus being treated by a community agency or family physicians.

In addition to demographic and clinical information, data on ACEs have been collected routinely since 2016 from a cohort of children and adolescents accessing addiction and mental health services within Calgary by CAAMHPP across its continuum of care [9]. The ACE questionnaire is administered by CAAMHPP clinicians using the ACE checklist through a face-to-face interview with children/adolescents aged below 18 years who seek specialty mental health care or with the parents if the children could not respond [42]. Preschool-aged children were also included in the study, and their ACE data were collected from the parents or caregivers. There is no predefined cut-off to determine whether the children or their parents would be the informant, and hence the clinicians administer it based on their clinical judgment. Informed consent was obtained from parents/guardians for the children below 18 years of age. The details about the ACE survey at CAAMHPP, such as data collection, storage, staff training, are described elsewhere [43]. The prospective follow-up of subsequent ED visits was available from the NACRS, a clinical, administrative database that collects all ambulatory care data, including ED visits within Canada [27]. The linkage was done using a common identifier, Personal Health Number (PHN), and the final analysis was performed using the linked and de-identified dataset.

The sample was comprised of children and adolescents aged 0 to 17 years who have enrolled in the AHS’s CAAMHPP program (intensive inpatient and outpatient mental health services) between June 2016 to June 2018 and all-cause ED visits during the year following their first registration. The end of the follow-up period was 30 June 2019. The linkage resulted in 6100 unique observations and 2545 matched ED visits within the year following enrollment.

### Inclusion and exclusion criteria

The study included all the patients aged 0–17 years registered in the RAIS database from June 2016 to June 2018. A single patient might have had multiple enrolments into the CAAMHPP program, but we included only the first enrolment since it is clinically more relevant to predict future outcomes at the point of the first contact with the program. Hence the ACE data used for prediction was the very first ACE assessment during the first enrollment. Also, a person might have had multiple ED visits, but we included only the index visit as per our operational definition. The ED visit on the same day of enrollment into the program was not considered an outcome because such predictions would not be useful for prevention.

### Measures

#### Predictors

##### ACE items

The 10 ACE items, namely exposure to physical abuse, witnessing inter-parental violence, parental divorce, parental substance use, emotional abuse, emotional neglect, physical neglect, incarcerated family member, and living with a mentally ill family member [9], were included as factor variables (yes/no/missing).

##### Demographic and clinical variables

Age at program enrollment was included as a continuous variable. Other categorical variables were reclassified and included as factor variables, which were namely sex (male; female; and neither male nor female), residence (urban; rural/unclassified or unknown; and missing), and program type (community bed; community clinic; consultation service; day treatment; emergency service; inpatient unit; outpatient service; school-based service; and specialized service). The factor variable primary diagnosis included categories: anxiety, bipolar disorder, depressive disorder, disruptive/impulse control/conduct disorder, feeding/eating disorder, gender dysphoria, medical diagnosis, neurodevelopmental disorder, obsessive-compulsive disorder, personality disorder, psychosocial factor, schizophrenia/psychotic disorder, somatic symptom, substance-related disorder, trauma/stressor-related disorder, other mental disorders, and others.

#### Outcome

The outcome was an ED visit (yes/no), defined as the index ED visit between 1 day to one-year post-first enrollment into the CAAMHPP services.

### Data management

**Fig. 1:**
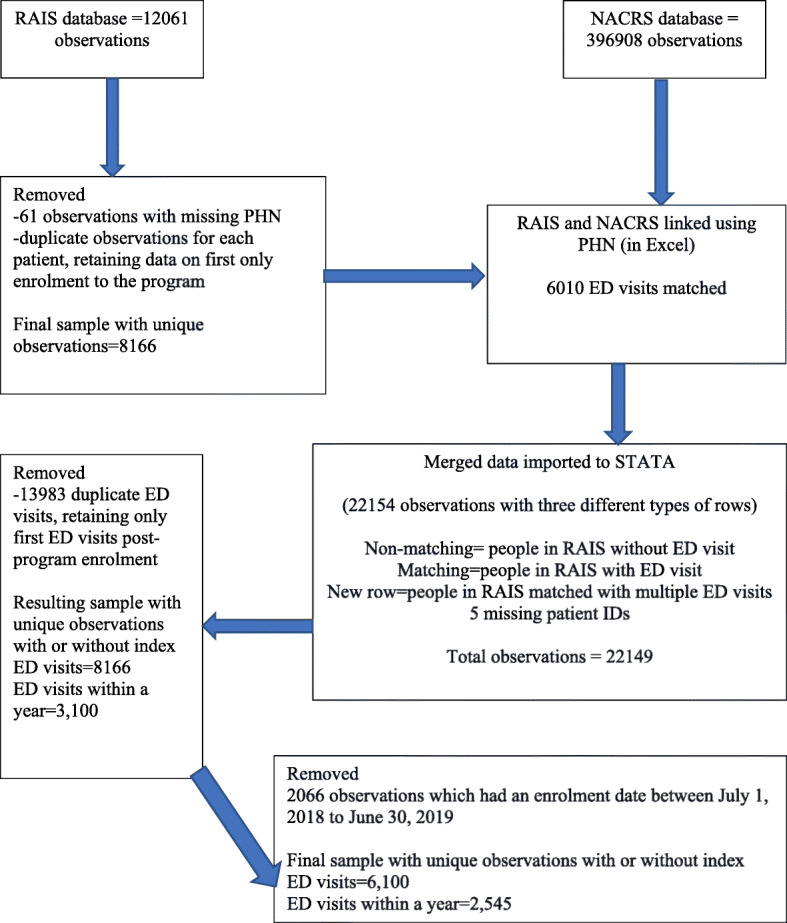
Derivation of the study sample

The encrypted RAIS and NACRS databases were made available by Alberta Health Services (AHS) through a secure data transfer process. The original data was available in Microsoft Excel format. The linkage was performed in Excel using a common identifier, Personal Health Number (PHN). The final linked de-identified dataset with 6100 unique observations (children enrolled in RAIS) matched with 2545 index ED visits (between 1 day to one-year post-enrollment to CAAMHPP) was extracted for analysis. The details of the data management process performed in Excel and STATA before reaching the final sample size from the raw data received is illustrated in the flow diagram (Fig. 1). All the analyses were performed in STATA/SE version 16 [44]. All the methods were carried out in accordance with University of Calgary’s guidelines and regulations for conducting research.

### Handling of missing data

The missing values in the predictor variables were less than 10% of the total sample. We examined the pattern of missing data in predictor variables in relation to the ED visit status (Table 2) and found that the missing data is consistently more among those with an ED visit than their counterparts. Hence, with an assumption of missing not at random (MNAR) and that the missing values may contribute to prediction [45], missing data were analyzed as a separate category. The assumption is well supported by the results (Table 3), i.e., higher odds of ED visits were seen among people not responding compared to those who reported no exposure in most of the predictor variables.

### Statistical analysis

#### Descriptive analysis

The subjects’ basic demographic and clinical characteristics were described as percentages and 95% Confidence Intervals (CIs). The prevalence of specific childhood adversities, all-cause, and mental illness specific index ED visits were also reported as percentages and 95% CIs. The distribution of the predictors was also stratified by ED visit status.

#### Measures of association

The association between childhood adversities and ED visits was examined using logistic regression analysis and the results are reported as Odds Ratios (OR) and 95% CIs.

#### Prediction modeling

##### Model development

The LASSO approach taken is a logistic regression with a regularization approach that incorporates a penalty to the log-likelihood function to shrink imprecise coefficients towards zero, thereby minimizing overfitting. The predictor selection is data driven. LASSO regression allows all predictors in the dataset in a single model, and predictor selection happens during the model development phase. The algorithms shrink the estimates (odds ratios) towards the null with shrinkage with the threshold for dropping a variable being governed by a single hyperparameter called lambda. Cross-validation (in the current study, 10-fold cross-validation) is used to identify the lambda value that produced the best-fitting parsimonious model, minimizing the model deviance [33, 45, 46]. LASSO is believed to have a crucial advantage of consistently identifying the true underlying model and effective handling of multicollinearity between predictors enabling navigation of complex associations in health care data [47, 48]. The dataset was split into two randomly selected sub-samples containing 75% and 25% of the total sample and named as “training” and “validation” samples. Splitting allows for a more accurate determination of the model’s performance in a new dataset and reduces overfitting [48]. The training sample was used to develop and internally validate the prediction models, and the validation sample was used to validate the selected prediction models externally.

Using the training data, two LASSO logistic regression models, each employing a different method to tune the shrinkage parameter lambda, namely Cross-validated (CV) and Adaptive, and performing 10-fold cross-validation for a set of 100 lambdas; were examined. CV is the default method of selecting tuning parameters in LASSO in STATA, which finds the lambda that minimizes the out-of-sample prediction error [49]. The adaptive method is a multistep version of the LASSO, the first step being CV and the second being CV among the covariates selected in the first step, also enabling exclusion of some extra variables [40, 44]. Each item in the ACEs checklist was treated as a separate predictor. ACE ratings have often been evaluated using simple scoring procedures (e.g., the number of reported adversities) using a cut-point based interpretation. However, some adversities are expected to have a greater impact than others, which is neglected in this approach to scoring. Regression-based machine learning techniques are capable of handling more complex data structures as these employ strategies to prevent over-fitting, thereby potentially making better use of the available data [50], and to reflect each item’s varying contribution. In the logistic regression-based machine learning approach used here, the regression coefficients associated with each adversity allow each one to make a different level of contribution to prediction. In total, ten ACE items, age, sex, residence, clinical diagnosis, program type, and the two-way interaction between each of the variables were included in the models to determine a model with the best predictive performance.

##### Assessment of model performance

The model performance was evaluated using measures of model discrimination and calibration. Discrimination of the model, which enables the model to separate individuals who end up to ER from those who do not, was quantified with sensitivity, specificity, positive and negative predictive value (PPV and NPV), and area under the receiver operating characteristics curve (AUC). The AUC ranges from 0 to 1, and the higher the AUC (preferably > 0.8), the better the prediction power of the model to be applied clinically [34, 36]. The resulting confusion matrices were used to visualise and identify the model maximizing correct classification (Accuracy = (True positive + True negative) / (True Positive + True Negative + False Positive + False Negative)). The overall average correct classification was also used to assess model performance. However, our goal was to attain a good balance between higher AUC, sensitivity, and PPV. The discrimination was assessed in both training and validation sub-sample, but with greater emphasis on the models’ performance in the validation sample.

Calibration of the model was assessed to measure the accuracy by which the model’s predicted event rates match the overall observed rates in the validation dataset. The predicted probability of ED visits was divided into four risk categories (< 25%, 25–50%, 50–75, and > 75%), and the relative frequency of observed events was calculated in each of those risk categories. If the model has good calibration, the empirical probability should be close to the predicted probability [51]. The risk stratification capacity was calculated as the proportion of children and adolescents categorized as low-risk or high-risk [29]. The calibration was also visualized by plotting observed ED visits in the x-axis vs. predicted ED visits in the y-axis, where the perfect predictions fall along the 45-degree line [45]. Additionally, the models’ predictive performance regarding out-of-sample prediction was assessed with an out-of-sample deviance ratio (value usually ranges from 0 to 1, but can be negative sometimes), which is the goodness of fit statistic. The out-of-sample deviance ratio in logistic LASSO models is analogous to the R-squared (higher the better; preferably more than 0.5) in the linear LASSO models, which refers to the proportion of variance in the outcome variable accounted for by the model under evaluation [49].

#### Subgroup analysis

Further subgroup analysis in terms of the LASSO models’ performance in predicting all-cause ED visits using ACE items was conducted among adolescents (10 to 17 years) separately. This subgroup is important because adolescence is a period that is more vulnerable in terms of ACEs exposure as well as mental illnesses [52]. The preventive care needs, and approaches might also be different between children and adolescents. Also, with the assumption that ACE items and the clinical and demographic information might be better predictive of cause-specific ED visits, we explored the predictive performance for mental health issues-specific ED visits among children and adolescents. All of the diagnoses listed in Table 1 except medical conditions were included as mental health issues. This approach is important because these prediction models’ utility is more for the health care providers working with children and adolescents with pre-existing mental health concerns at the CAAMHPP program. They are thus more concerned with and are more equipped to implement strategies that prevent mental illness related ED visits if they could predict such visits ahead of time.

**Table 1:** Distribution of the demographic and clinical characteristics in the full sample and across ED visit status

Characteristics	Full sample % (95% CI)	ED visit % (95% CI)	No ED visits % (95% CI)
Age in years (mean/SD)	12.1 (4.3)	12.6 (4.2)	11.7 (4.3)
Sex			
Male	44.7 (43.5,46.0)	40.4 (38.5,42.4)	47.8 (46.2,49.4)
Female	54.2 (53.0,55.5)	58.2 (56.3,60.1)	51.4 (49.7,53.0)
Neither male/female	1.1 (0.8,1.4)	1.4 (1.0,1.9)	0.8 (0.6,1.2)
Residence			
Rural	4.8 (4.3,5.4)	3.5 (2.9,4.3)	5.8 (5.0,6.6)
Urban	89.5 (88.7,90.3)	91.8 (90.7,92.8)	87.9 (86.8,89.0)
Unclassified/unknown	0.8 (0.6,1.0)	0.6 (0.4,1.0)	0.9 (0.6,1.2)
Missing	4.9 (4.4,5.4)	4.1 (3.4,4.9)	5.4 (4.7,6.2)
Program type			
Community bed	1.3 (1.1,1.6)	1.8 (1.4,2.4)	1.0 (0.7,1.3)
Community clinic	22.2 (21.2,23.3)	21.6 (20.0,23.2)	22.7 (21.3,24.1)
Consultation service	10.5 (9.7,11.3)	10.8 (9.7,12.1)	10.2 (9.3,11.3)
Day treatment	1.4 (1.1,1.7)	1.2 (0.9,1.7)	1.5 (1.2,2.0)
Emergency service	14.5 (13.6,15.4)	17.6 (16.2,19.1)	12.2 (11.2,13.3)
Inpatient unit	13.7 (12.8,14.5)	17.7 (16.3,19.3)	10.7 (9.8,11.8)
Outpatient service	16.3 (15.4,17.3)	15.3 (14.0,16.8)	17.0 (15.8,18.3)
School-based service	14.6 (13.7,15.5)	9.0 (8.0,10.2)	18.5 (17.3,19.8)
Specialized service	5.6 (5.0,6.2)	4.9 (4.1,5.8)	6.1 (5.3,6.9)
Primary diagnosis			
Anxiety disorder	24.3 (23.2,25.4)	23.5 (21.9,25.1)	24.9 (23.5,26.3)
Bipolar disorder	0.2 (0.1,0.4)	0.2 (0.1,0.5)	0.2 (0.1,0.4)
Depressive disorder	12.5 (11.7,13.4)	15.0 (13.6,16.4)	10.8 (9.8,11.8)
Disruptive/impulse-control/conduct disorder	1.5 (1.2,1.8)	1.8 (1.3,2.4)	1.3 (0.9,1.7)
Feeding/eating disorder	2.4 (2.0,2.8)	2.2 (1.7,2.8)	2.5 (2.1,3.1)
Gender dysphoria	0.2 (0.1,0.3)	0.2 (0.1,0.5)	0.2 (0.1,0.4)
Medical disorder	0.7 (0.5,0.9)	0.7 (0.4,1.1)	0.7 (0.5,1.0)
Neurodevelopmental disorder	19.5 (18.5,20.5)	19.3 (17.8,20.9)	19.7 (18.4,21.0)
Obsessive-compulsive disorder	1.6 (1.3,2.0)	1.1 (0.8,1.6)	2.0 (1.6,2.5)
Other mental disorders	4.2 (3.8,4.8)	3.3 (2.7,4.1)	4.9 (4.2,5.7)
Personality disorder	0.2 (0.1,0.4)	0.2 (0.1,0.5)	0.3 (0.1,0.5)
Psychosocial factor	17.3 (16.4,18.3)	14.7 (13.4,16.2)	19.1 (17.9,20.5)
Schizophrenia/psychotic disorder	0.7 (0.5,1.0)	0.9 (0.6,1.4)	0.6 (0.4,0.9)
Somatic symptom	0.3 (0.2,0.5)	0.6 (0.3,0.9)	0.2 (0.1,0.4)
Substance-related disorder	0.9 (0.7,1.2)	1.2 (0.9,1.7)	0.7 (0.5,1.0)
Trauma/stress-related disorder	13.1 (12.3,14.0)	14.8 (13.5,16.2)	12.0 (10.9,13.1)
Others	0.2 (0.1,0.3)	0.3 (0.2,0.6)	0.1 (0.0,0.3)

#### Sensitivity analysis

There is no consensus on the way ACEs are evaluated, and all the approaches being used have their own value. Apart from the effect of individual ACE items on health, the dose-response relationship between ACEs and adverse health has also been consistently reported in research. The ACEs as a cumulative score examine the adversities as a single construct, irrespective of the specific adversities involved, and address the co-occurrence of such adversities [53]. We examined whether examining ACEs as a cumulative score improves the predictive performance. Also, rather than creating a single cumulative score, some researchers have advocated categorizing the exposure into separate groups based on the types of ACEs, taking into consideration the differential mechanism towards their psychopathological sequelae [54]. Based on the dimensional model of adversity suggested in the existing literature [53, 54], we examined the predictive ability of threat based (emotional abuse, sexual abuse, physical abuse, and witnessing intimate partner violence) and deprivation based (emotional neglect, physical neglect, parental divorce, mental illness in family, substance use in family and incarceration of family member) adversities from the ACEs checklist in two separate models.

## Results

### Descriptive

The total sample size in the linked data was 6100. The description of the cohort of children and adolescents understudy with stratification by ED visit status is presented in Table 1. The average age of the sample was 12.1 (SD 4.3) years, ranging from 0 to 17 years. The majority of the participants were females (54.2%), and most resided in urban areas (89.5%). Anxiety was the most prevalent disorder (24.3%) at admission to the mental health services, followed by neurodevelopmental disorders (19.5%), psychosocial factors (17.3%), trauma/stressor-related disorders (13.1%), and depressive mood disorders (12.5%). Most of the participants were enrolled from community clinics (22.2%), followed by outpatient services (16.3%), emergency services (14.5%), and school-based services (14.6%).

The prevalence of a one-year index all-cause ED visit in the sample was 41.7% (95% CI 40.5, 43.0). The mean number of ED visits within a year, among those making at least one ED visit, was 3.4 (SD 4.1) times. Most of the index ED visits were for medical conditions (59.1%), followed by depressive disorder (10.3%), trauma-stress-related disorder (9.9%), and anxiety disorder (6.0%). The most-reported ACE items were living with a mentally ill family member (50.0%) and parental divorce (48.7%), and the least reported were sexual abuse (6.8%) and having an incarcerated family member (7.8%). Compared to those without ED visits, more children and adolescents with ED visits reported exposure to individual ACE items (Table 2).

**Table 2:** Distribution of reports of ACE items the full sample and across ED visit status

Characteristics	Full sample % (95% CI)	With ED visit % (95% CI)	Without ED visit % (95% CI)
Emotional abuse			
Yes	26.5 (25.4,27.6)	30.9 (29.1,32.7)	23.3 (22.0,24.8)
No	71.1 (70.0,72.2)	66.5 (64.6,68.3)	74.4 (73.0,75.8)
Missing	2.4 (2.0,2.8)	2.6 (2.1,3.3)	2.2 (1.8,2.8)
Physical abuse			
Yes	13.6 (12.8,14.5)	16.6 (15.2,18.1)	11.4 (10.4,12.5)
No	83.4 (82.5,84.3)	80.0 (78.4,81.5)	85.9 (84.7,87.0)
Missing	3.0 (2.6,3.4)	3.4 (2.8,4.2)	2.7 (2.2,3.3)
Sexual abuse			
Yes	6.8 (6.2,7.5)	8.8 (7.8,10.0)	5.4 (4.7,6.2)
No	89.2 (88.4,90.0)	86.2 (84.9,87.5)	91.4 (90.4,92.2)
Missing	3.9 (3.5,4.5)	5.0 (4.2,5.9)	3.2 (2.7,3.8)
Emotional neglect			
Yes	29.9 (28.7,31.0)	34.3 (32.5,36.2)	26.7 (25.3,28.2)
No	67.1 (65.9,68.3)	62.9 (61.0,64.7)	70.1 (68.6,71.6)
Missing	3.0 (2.6,3.5)	2.8 (2.2,3.5)	3.2 (2.7,3.8)
Physical neglect			
Yes	11.2 (10.5,12.0)	12.7 (11.5,14.1)	10.2 (9.2,11.2)
No	86.3 (85.4,87.2)	84.6 (83.1,85.9)	87.6 (86.4,88.6)
Missing	2.4 (2.1,2.9)	2.7 (2.1,3.4)	2.3 (1.8,2.8)
Parents divorced			
Yes	48.7 (47.5,50.0)	51.6 (49.7,53.6)	46.6 (45.0,48.3)
No	50.3 (49.1,51.6)	47.2 (45.3,49.2)	52.6 (50.9,54.2)
Missing	0.9 (0.7,1.2)	1.1 (0.8,1.6)	0.8 (0.5,1.1)
Interpersonal violence in family			
Yes	19.4 (18.4,20.4)	21.9 (20.3,23.5)	17.6 (16.3,18.8)
No	76.5 (75.4,77.5)	73.2 (71.5,74.9)	78.8 (77.4,80.1)
Missing	4.1 (3.7,4.7)	4.9 (4.1,5.8)	3.6 (3.1,4.3)
Substance use in family			
Yes	25.0 (24.0,26.1)	27.0 (25.3,28.8)	23.6 (22.2,25.0)
No	70.7 (69.5,71.8)	67.9 (66.1,69.7)	72.7 (71.2,74.2)
Missing	4.3 (3.8,4.8)	5.1 (4.3,6.0)	3.7 (3.1,4.4)
Mental illness in family			
Yes	50.0 (48.7,51.2)	52.3 (50.3,54.2)	48.3 (46.7,50.0)
No	46.5 (45.2,47.7)	43.7 (41.8,45.7)	48.4 (46.8,50.1)
Missing	3.6 (3.1,4.1)	4.0 (3.3,4.8)	3.3 (2.7,3.9)
Family member in prison			
Yes	7.8 (7.1,8.5)	7.9 (6.9,9.0)	7.7 (6.9,8.6)
No	86.3 (85.4,87.1)	84.7 (83.3,86.1)	87.4 (86.3,88.5)
Missing	5.9 (5.4,6.6)	7.4 (6.5,8.5)	4.9 (4.2,5.6)

Most of the ACE items (except incarcerated family members) were significantly associated with index ED visits within a year, as evidenced by a p-value of < 0.05 and non-inclusion of the null value of OR = 1 within the 95% CI. The statistically significant odds ratios ranged from 1.20 to 1.72, suggesting a moderate association (Table 3).

**Table 3:** Association between ACE items and index ED visit within a year of enrolment to mental health services

ACE items	Odds Ratio (OR)	95% CI	***p***-value
Emotional abuse			
Yes	1.48	1.32, 1.66	< 0.001
No	Ref	Ref	Ref
Missing	1.33	0.95, 1.85	0.09
Physical abuse			
Yes	1.56	1.35, 1.81	< 0.001
No	Ref	Ref	Ref
Missing	1.37	1.02, 1.85	0.03
Sexual abuse			
Yes	1.72	1.41, 2.10	< 0.001
No	Ref	Ref	Ref
Missing	1.64	1.26, 2.12	< 0.001
Emotional neglect			
Yes	1.43	1.28, 1.60	< 0.001
No	Ref	Ref	Ref
Missing	0.97	0.72, 1.31	0.8
Physical neglect			
Yes	1.30	1.11, 1.52	0.001
No	Ref	Ref	Ref
Missing	1.21	0.88, 1.68	0.2
Parents divorced			
Yes	1.23	1.11, 1.37	< 0.001
No	Ref	Ref	Ref
Missing	1.61	0.95, 2.72	0.07
Interpersonal violence in family			
Yes	1.34	1.18, 1.53	< 0.001
No	Ref	Ref	Ref
Missing	1.44	1.12, 1.86	0.004
Substance use in family			
Yes	1.23	1.09, 1.38	0.001
No	Ref	Ref	Ref
Missing	1.47	1.15, 1.89	0.002
Mental illness in family			
Yes	1.20	1.08, 1.33	0.001
No	Ref	Ref	Ref
Missing	1.36	1.03, 1.79	0.02
Incarceration of family member			
Yes	1.05	0.87, 1.27	0.6
No	Ref	Ref	Ref
Missing	1.57	1.27, 1.95	< 0.001

### LASSO logistic regression models

The summary and goodness-of-fit statistics for the LASSO models in the training data are shown in Table 4. Refer to Additional file 1 for the list of variable combinations with non-zero coefficients retained by the two LASSO models.

**Table 4:** LASSO model summary statistics and goodness-of-fit statistics in predicting all-cause ED visits in children and adolescents

Model	lambda	Number of non-zero coefficients	In-sample deviance ratio	Out of sample deviance ratio
Cross Validated	0.0107871	113	0.066	0.0246
Adaptive	0.0016839	104	0.099	−0.0448

#### Discrimination

Table 5 represents the discriminative ability of the LASSO models. The average cross-validated AUC, sensitivity, specificity, PPV, NPV, and overall classification accuracy within the training dataset and the same calculated in the external validation dataset are presented. None of the models were significantly superior in their discrimination ability, both having an AUC around 0.6. This suggests a 60% probability that a randomly chosen individual with an ED visit will have a higher risk score than a randomly selected individual without the outcome. Using the classification threshold of 0.5, the CV model achieved a sensitivity of 29.2%, PPV of 52.5%, and overall classification accuracy of 60.9% in the validation dataset. The adaptive model appeared to have a better balance between AUC, sensitivity, and accuracy. The sensitivity achieved from the adaptive model was 37.8%, the PPV was 49.6%, and the overall classification accuracy was 59.5%. We saw a slightly weaker performance of the CV model in the validation dataset (AUC in training dataset 0.680–0.698 vs. AUC in validation dataset 0.610–0.604), which is expected. However, the large difference between the AUCs in training and validation datasets (0.698 vs. 0.604) in the adaptive model suggests that the model was overfitting. We set different classification thresholds to see whether the models attain better discrimination than those with a threshold of 0.5; however, there were no meaningful improvements (results not shown).

**Table 5:** Discrimination performance of the LASSO logistic regression models in the validation dataset in predicting all-cause ED visits among children and adolescents

Model	Training data	Validation data					
	AUC	AUC	Sensitivity%	Specificity%	PPV%	NPV%	Overall %
Cross Validated	0.680	0.610	29.2	82.2	52.5	63.3	60.9
Adaptive	0.698	0.604	37.8	74.1	49.6	63.9	59.5

**Fig. 2:**
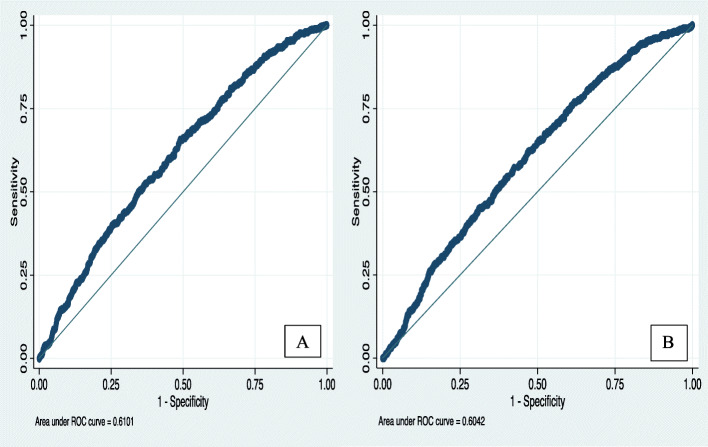
ROC curves of the LASSO logistic regression models in validation dataset in predicting all-cause ED visits among children and adolescents (**A**) CV (**B**) Adaptive

The two models’ discrimination is illustrated by the graph of ROC curves (plotted as sensitivity vs. (1-specificity)) in Fig. 2. Both the curves, i.e., from the CV and adaptive model, were close to the reference line.

#### Calibration

The calibration performance of the models in the validation dataset is represented in the calibration matrix in Table 6. A good calibration refers to less variability between the observed and predicted rates of ED visits. It is also more acceptable if the model under-triages low-risk individuals than if it under triages high-risk individuals. In the adaptive model, the observed rate and 95% CI (39.8, 95%CI 36.5, 43.1) fell within the 25–49% risk category, whereas there was over-triage in the low risk (predicted probability < 25%) category and under-triage in the high risk (predicted probability > 50%) categories. The variability was highest in the 75–100% risk category, observed ED visits 43.1% (95% CI 32.0, 54.9). In the CV model, the observed rates and corresponding 95% CI fell within the < 25% and 25–49% risk categories but failed to do so in the higher-risk categories where there was evidence of under-triage. The models assigned a small number of predicted probabilities for the highest and lowest risk children and adolescents. The adaptive model had slightly higher risk stratification capacity in the lowest and highest risk categories (4.2 and 15.7%, respectively). The out-of-sample deviance ratio of the CV and adaptive models were far less than 1 (0.0246 and − 0.0448, respectively) (Table 4).

**Table 6:** Calibration matrix of the LASSO regression models in the validation dataset in predicting all-cause ED visits among children and adolescents

Model	Predicted probability of ED visit %	Observed ED visit % (95%CI)	Risk stratification capacity N (%)
Cross Validated	0–24	15.0 (9.2,23.5)	393 (6.44)
	25–49	38.8 (35.9,41.7)	4330 (70.98)
	50–74	52.5 (47.2,57.8)	1364 (22.36)
	75–100	50.0 (4.0,96.0)	13 (0.21)
Adaptive	0–24	21.7 (16.6,27.8)	957 (15.69)
	25–49	39.8 (36.5, 43.1)	3296 (54.03)
	50–74	50.8 (45.8,55.7)	1592 (26.10)
	75–100	43.1 (32.0, 54.9)	255 (4.18)

**Fig. 3:**
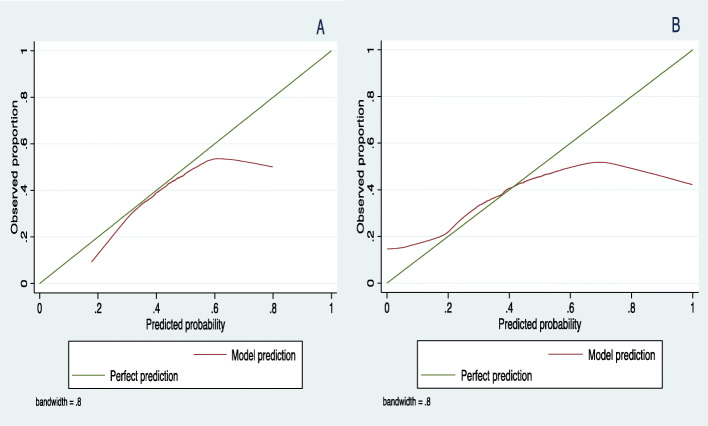
Calibration plots of the LASSO models in validation dataset in predicting all-cause ED visits among children and adolescents; (**A**) CV (**B**) Adaptive

The LASSO models’ calibration performance is illustrated by the observed versus predicted values plot in Fig. 3 (A, B). There is good calibration if the model predictions curve is closer to the perfect prediction line. The plot for the CV model (Fig. 3A) shows that the predicted probabilities are lower on average than observed, especially in the low and high-risk levels. The adaptive model plot (Fig. 3B) shows that the predicted probabilities are higher than those observed in lower-risk groups and lower than those observed in higher-risk groups.

Since none of the models had a good predictive performance, we did not perform additional inferential analysis with the variables retained by the models and hence no odds ratios are presented here based on LASSO models. Also, the coefficients selected by the two models are not presented and discussed here since the models did not have an adequate predictive performance for the coefficients to be usable in creating risk-scoring algorithms.

### Subgroup analysis

Within a year of enrolment to the CAAMHPP program, 16.8% (95%CI 15.9, 17.8) of children and adolescents visited the ED with mental health issues. The CV and adaptive LASSO models failed to perform well (poor discrimination and calibration) in predicting mental illness specific ED visits among children and adolescents (Supplementary Tables 1 and 2: Additional file 2). The sample comprised 75.3% adolescents, and 45% among them had an ED visit within a year. The two LASSO models did not demonstrate adequate performance in predicting all-cause ED visits among adolescents as well (Supplementary Tables 3 and 4: Additional file 2).

### Sensitivity analysis

The median ACE score and interquartile range in the sample was 2 (IQR 1-4). We examined the ability of the cumulative scores in predicting ED visits in our study and found that the performance was inadequate (AUC < 0.7) (Supplementary Tables 1 and 2: Additional file 3). The predictive ability of threat-based and deprivation-based adversities from the ACEs checklist examined as two separate models also failed to demonstrate clinically meaningful predictive performance as evidenced by AUC < 0.7 (Supplementary Tables 3 to 6: Additional file 3).

## Discussion

### Summary

The study’s objective was to explore whether predictive models using machine learning approaches could predict ED visits with sufficient accuracy to move forward with developing a decision support tool for clinicians to stratify children and adolescents into various risk categories.

The bivariate analysis in the full sample showed that most of the ACE items were significantly associated with the subsequent ED visit. The statistically significant odds ratios ranged from 1.2 to 1.7; for instance, the odds ratio associated with sexual abuse was 1.72 (95% CI 1.41,2.10), with physical abuse was 1.56 (95% CI 1.35,1.81) and with parental divorce was 1.23 (95% CI 1.11,1.37). This finding is consistent with the previous literature, which has reported that ACEs are associated with higher health service utilization [10, 11, 13, 14]. The strong association of the adversities with ED visits indicates the important role of ACEs in health care utilization, even if they do not accurately support individual level predictions. Other demographic and clinical characteristics such as being female and having a mental health diagnosis were associated with frequent ED visits among children and adolescents in the study, which is also similar to the findings of other studies [19, 20].

However, the prediction of outcomes at the individual level is much more challenging than comparing frequencies according to statistical significance. The LASSO logistic regression models employing two different lambda selection methods, namely cross-validated and adaptive, failed to perform well in the database in predicting ED visits. The AUCs never reached 0.80 in the validation sample to be considered good discriminatory performance, and the ROC curves were closer to the reference line. This means that the models did not discriminate well between children who did and did not visit ED. The maximum sensitivity achieved was 37.8%, which means that the model identified only 37.8% of the children who actually visited ED, and the PPV was 49.6%, which means that of those who were predicted or flagged by the model to have an ED visit in future, not even 50% of them had an ED visit and more than 50% would be false positive. A good calibration performance is more important when the purpose of prediction is prognostic [35]. Concordance between observed and predicted rates of ED visits was not achieved in the various risk categories (< 25%, 25–49%, 50–74%, > 75%) to be considered a good calibration performance, where there was evidence of over-triage in the low-risk category and under-triage in the high-risk category. The poor calibration was evident in the graphs of predicted versus observed proportions, where the fitted calibration curves deviated substantially from the reference/perfect prediction line. The out-of-sample deviance ratios were far less than 1, demonstrating their inability to perform well in the validation dataset. Hence, the ACE items in the ACE checklist did not have a useful degree of ability to identify children and youth at risk of requiring a visit to the ED within a year of presenting to the mental health services and thus cannot be applied clinically to stratify risk of ED visits.

The subgroup analysis showed that ACEs did not perform well in predicting all-cause ED visits among adolescents and predicting mental-illness-related ED visits among children and adolescents. Furthermore, the prediction models did not show promising results in predicting ED visits in an earlier period, i.e., within 6 months of enrolment to the program (results not shown). Similarly, the use of cumulative ACE scores and different dimensions of adversities (threat and deprivation) also did not improve the predictions. The adaptive LASSO prediction model using ACE scores had an AUC of 0.621 with a sensitivity of 38.9%. The model with threat-based adversities had an AUC of 0.608 (sensitivity 38.1%), and that with deprivation-based adversities had an AUC of 0.607 (sensitivity 38.6%). These discrimination properties were similar to those achieved using each ACE item separately in the model (AUC 0.604, sensitivity 37.8%). There was non-concordance between observed and predicted rates of ED visits in all the models. This suggests that the predictive performances of the various approaches of modeling adverse childhood experiences used in the study were equivalent in predicting ED visits among children and adolescents with pre-existing mental health issues.

### Interpretation

If the prediction model had performed well, it might have been sufficiently accurate for the clinicians and policymakers to early identify which of the patients would be visiting the ED for physical or mental health complaints in the future. This valuable information might have supported the development of decision support tools or improvements to care pathways in outpatient services to prevent ED visits through improved patient education, discharge planning, and follow-up care. Also, there are many resources required to collect and store data in a clinical setting and collecting ACEs data has challenges due to the sensitive nature of the subject. Hence, the policymakers and care providers need to know whether the data on ACEs should be routinely collected or not by clinicians. The prediction models are not intended to replace clinicians but support them in their clinical judgment by combining knowledge of machine learning with knowledge about childhood exposure to adversity [55]. The prediction models formulated by incorporating a wide range of predictors provide them additional information that they might not have access to or might not have enough time and resources to take into account while formulating their decisions, making the care pathways more efficient. Health policymakers and administrators are always looking for ways to use scarce resources efficiently, and prediction models could help do so [31]. However, such prediction models should be developed and used with caution because the use of poor-performing models to predict events and guide decisions may, in turn, harm the clients as well as waste the scarce resources.

While the predictive models developed in this project ultimately did not predict ED visits with sufficient accuracy to be clinically useful, research of the type presented here is important to the current trend in the direction of precision health. The situations in which routinely collected data may have value for prediction and prediction may have value for clinical management are not well understood and need to be explored. The results presented here suggest that despite statistical associations of the ACEs with relevant clinical outcomes, the addition of ACE items provide insufficient data to predict ED visits at the individual level, which lacks utility for clinical use. Future studies, including a more extensive set of predictors such as the severity of mental illness, a wide range of other childhood adversities with their frequency and severity, age at exposure, other socio-demographic variables such as family income, parental education status, social support, resilience and other important clinical or behavioral information, are required which might improve the predictive ability of the machine learning models. These variables are reported to be associated with increased health service utilization [11, 19, 20]. However, a significant association with the outcome is not exclusive to being included as potential predictors in a prediction model.

The RAIS database contains a limited range of other variables that could improve the models’ predictive ability. Hence, child and adolescent mental health programs should consider collecting more information about childhood exposure to adversities. A recent Canadian study has suggested that in addition to the ACE items, expanded ACEs such as household gambling problems, spanking, peer victimization, and neighborhood safety factor well with child maltreatment and household dysfunction and are associated with poor physical and mental health [56]. Collecting data on these emerging adversities through the health system and using them to predict health outcomes could also be the future direction.

Our results do not suggest that ACEs are not useful or not important for prediction at all. Although the vast majority of literature which reports child maltreatment as predictors of adverse health have merely examined associations [25, 57, 58], few studies have used ACEs to predict health outcomes using a prediction modeling approach. The limited studies have found that history of child abuse and household dysfunctions predict adverse mental health and psychosocial disadvantages [35, 59, 60], but not much is published about predicting health service utilization. The outcome, i.e., ED visits, maybe inherently less predictable as it may depend on chance events such as injuries, illness, or psychosocial circumstances such as homelessness and poverty [19]. Also, ACEs might be used for many purposes other than predicting ED visits. These include health risk behaviors, chronic diseases, and hospitalizations, and future studies should look into such predictions. The ability to predict and mitigate other proximal adverse health effects of ACEs such as smoking, alcohol and illicit drug use may also benefit children and adolescents to prevent future health emergencies and unplanned need for acute health care services [47].

The population under study was a highly selected subgroup (i.e., children who are already seeking mental health services), which is already a high-risk cohort for ED visits. Future studies should also explore whether ACE items could predict ED visits among children and adolescents from the general population. The ACE checklist in itself has its limitations in terms of limited item coverage and has not been validated [61]. Hence, the inability of the ACE checklist to demonstrate good predictive ability does not suggest that other validated tools that measure childhood adversities, such as Childhood Trauma Questionnaire (CTQ), Conflict Tactic Scales, may not be useful in predicting health outcomes.

The LASSO models’ poor performance in the dataset we used may not mean that machine learning lacks utility for predicting health outcomes. It has been reported that machine learning techniques demonstrated good performance in predicting adverse health that may develop after child abuse [59]. Most of our predictors were categorical variables, and some reports suggest that machine learning might perform better in prediction if there are a large number of continuous variables than binary or factor variables [47]. Furthermore, it is not surprising that the small number of variables we used were not adequately predictive of the outcome [47, 48]. We also used only one type of machine learning approach (i.e., LASSO regression) for predicting our outcome, which assumes a linear relationship between predictors and the outcome. Future studies should assess whether the ACE items predict future ED visits by employing other machine learning approaches such as neural networks, gradient boosted trees, and support vector machines, which can take into account the nonlinearities in the relationship between the predictors and the outcome better, if any [47]. The performance of the models may also improve with a bigger sample size [48], which in our case can be achieved by analyzing a more extended period of data.

The study has some strengths and limitations. This study’s relevance is strong because childhood adversities are a significant public health problem, leading to various health issues throughout the lifespan and contributing to an increased burden on health services and considerable healthcare costs. Prediction modeling is a new and advanced method of incorporating routinely available data into precision health applications. This study used routinely collected ACE data to predict future ED visits among children and adolescents for the first time in Alberta Health Services. The study data came from patients from one geographic region of Alberta; the prediction modeling results might not be generalizable to other populations due to differences in the prevalence of the outcome or inclusion and exclusion criteria of the patient population. However, the generalizability has low priority in prediction modeling because the models are generally optimized for a particular clinical setting to aid in clinical judgment. The random split sample for training and validation and cross-validation method applied in the LASSO models might have replication instability such that the different random subsets of data might lead to differences in the prediction performance measures [45]. However, the difference was not big enough to change the interpretation of the results in this study. The parental reports of ACEs might have been biased (potentially under-reported) due to the stigma attached to the adversities, the fear of child welfare reporting, or being the perpetrators themselves. However, the ACEs were still significantly associated with ED visits. Hence, we do not believe that the heterogeneity of the informants on ACEs would have affected the performance of prediction models. As described above, an indicator of missing data was included as a predictor in the modeling and contributed to the predictive algorithm. However, indicators for actual events rather than missing data indicators would have improved the predictive performance of the model to a greater extent. Therefore, missing data remains a limitation of this study. Although the objective of the study was to assess the predictive ability of ACE checklist, it is important to acknowledge that the checklist addresses a very limited range of possible childhood adversities that are more relevant to the population under study. For instance, the sample was predominantly urban, and it has been reported that urban youth are at increased risk of exposure to violence in community, both direct or indirect victimization, and the exposure would be higher if they belonged to poor and disadvantaged households and communities [62]. These exposures may increase the risk of adverse health and behavioural outcomes and might prove to be significant predictors of poor health and health service utilization in future studies. To further the goal of prediction, it will be important that such adversities be collected and recorded in accessible data sources.

## Conclusion

The ACE items from the ACE checklist were strongly associated with ED visits. However, the ACEs, along with the clinical and demographic information available from the RAIS database and their various combinations, did not demonstrate acceptable performance in predicting ED visits within a year of enrolment in an intensive outpatient mental health service program, and hence was not sufficient for application in clinical practice. This result was obtained using a state-of-the-art machine learning model with known strong predictive performance. We require good sensitivity and PPV in the model to correctly identify children who need additional interventions and not deploy resources inappropriately. The findings suggest that ACEs are important risk factors associated with health service utilization but cannot be viewed as deterministic causes of health events. Other machine learning methods or statistical models that include additional important variables such as the severity of mental illness, frequency and severity of ACEs, peer victimization, neighborhood safety, social support, and resilience may produce better performing prediction models. Such models can aid in clinical judgment, implementation of care pathways, and patient education to prevent ED visits. However, they would require a large quantity of additional routinely collected data, especially childhood adversity data in greater depth and breadth.

## Supplementary Information

**Additional file 1.** Coefficients selected by the LASSO models.

**Additional file 2.** Results of subgroup analyses.

**Additional file 3.** Results of sensitivity analyses.

## Data Availability

The datasets generated and/or analysed during the current study are not publicly available due to limitations of ethical approval involving the patient data and anonymity but are available from the corresponding author on reasonable request.

## Abbreviations

ACEs: Adverse Childhood Experiences
ED: Emergency Department
AHS: Alberta Health Services
CAAMHPP: Child and Adolescent Addiction and Mental Health and Psychiatry Program
RAIS: Regional Access and Intake System
NACRS: National Ambulatory Care and Reporting System
LASSO: Least Absolute Shrinkage and Selection Operator
AUC: Area Under the receiver Operating Characteristics Curve
CV: Cross-validated
PPV: Positive Predictive Value
