# Supplementary material for: Can the adverse childhood experiences (ACEs) checklist be utilized to predict emergency department visits among children and adolescents?
Source: BMC Med Res Methodol. 2021 Sep 25;21:195. doi: 10.1186/s12874-021-01392-w (PMC8465692; doi:10.1186/s12874-021-01392-w)
Supplement: Supplementary file 1 — Additional file 1. Coefficients selected by the LASSO models. [file 12874_2021_1392_MOESM1_ESM.docx]

# Can the Adverse Childhood Experiences (ACEs) checklist be utilized to predict emergency department visits among children and adolescents?

Asmita Bhattarai, MPH ^a, b^, Gina Dimitropoulos, PhD ^b, c^, Brian Marriott ^c, d^, Jaime Paget ^d^, Andrew G.M. Bulloch, PhD ^a, b, e^, Suzanne C. Tough ^a, f^, Scott B. Patten, MD, PhD ^a, b, e^

^a^ Department of Community Health Sciences, Cumming School of Medicine, University of Calgary, 3280 Hospital Drive NW, Calgary, AB, Canada. T2N4Z6.

^b^ Mathison Centre for Research & Education, University of Calgary, 3280 Hospital Drive NW, Calgary, AB, Canada. T2N4Z6.

^c^ Faculty of Social Work, University of Calgary, 2500 University Dr NW, Calgary, AB, Canada. T2N 1N4

^d^ Addiction and Mental Health, Alberta Health Services- Calgary Zone, Canada

^e^ Department of Psychiatry, Cumming School of Medicine, University of Calgary, 2500 University Dr NW, Calgary, AB, Canada. T2N 1N4.

^f^ Department of Pediatrics, Cumming School of Medicine, University of Calgary, 2500 University Dr NW, Calgary, AB, Canada. T2N 1N4.

# Corresponding Author:

Asmita Bhattarai, PhD Candidate (Epidemiology)

Department of Community Health Sciences, Cumming School of Medicine, University of Calgary, 3280 Hospital Drive NW, Calgary, AB, Canada. T2N4Z6.

(T) (587) 917-5060

(E) [asmita.bhattarai1@ucalgary.ca](mailto:asmita.bhattarai1@ucalgary.ca) (ORCID) 0000-0001-6689-7218

# Additional file 1: Coefficients selected by the LASSO models

**-------------------------------------------------------------------------------------**

**| crossvalidated adaptive**

**----------------------------------------------------------+--------------------------**

**final_emotionalabuse#final_sex |**

**yes#female | x x**

**|**

**final_emotionalabuse#final_diagnosis |**

**yes# depressive | x x**

**yes#personality | x x**

**no#bipolar | x**

**|**

**final_emotionalabuse#final_programtype |**

**no#community bed | x x**

**|**

**final_emotionalabuse#final_physicalabuse |**

**no#yes | x x**

**no#missing observations | x x**

**|**

**final_emotionalabuse#final_sexualabuse |**

**yes#missing observations | x x**

**|**

**final_emotionalabuse#final_physicalneglect |**

**no#missing observations | x**

**|**

**final_emotionalabuse#final_parentsdivorced |**

**no#missing observations | x x**

**|**

**final_emotionalabuse#final_ipv |**

**no#no | x x**

**yes#no | x x**

**|**

**final_emotionalabuse#final_fammentalillness |**

**missing observations#missing observations | x x**

**|**

**final_physicalabuse#final_residence |**

**yes#rural/mod urban inf/rural centre | x x**

**yes#urban/metro/mod metro inf | x x**

**|**

**final_physicalabuse#final_diagnosis |**

**no#other mental | x x**

**missing observations#psychosocial factor | x x**

**|**

**final_physicalabuse#final_programtype |**

**yes#community bed | x x**

**|**

**final_physicalabuse#final_emotionalneglect |**

**missing observations#yes | x x**

**|**

**final_physicalabuse#final_famsubsuse |**

**yes#yes | x x**

**|**

**final_physicalabuse#final_famprison |**

**no#yes | x x**

**no#missing observations | x x**

**|**

**final_sexualabuse#final_sex |**

**no#male | x x**

**|**

**final_sexualabuse#final_diagnosis |**

**yes#obsessive-compulsive | x x**

**missing observations#neurodevelopmental | x x**

**|**

**final_sexualabuse#final_programtype |**

**no#school based service | x x**

**missing observations#community clinic | x x**

**|**

**final_sexualabuse#final_physicalneglect |**

**yes#yes | x**

**|**

**final_sexualabuse#final_parentsdivorced |**

**yes#yes | x x**

**|**

**final_emotionalneglect#c.final_age |**

**yes | x x**

**|**

**final_emotionalneglect#final_residence |**

**yes#rural/mod urban inf/rural centre | x x**

**|**

**final_emotionalneglect#final_diagnosis |**

**no#other mental | x x**

**yes#obsessive-compulsive | x x**

**missing observations#neurodevelopmental | x x**

**missing observations#other mental | x x**

**missing observations#psychosocial factor | x x**

**|**

**final_emotionalneglect#final_programtype |**

**no#emergency service | x x**

**no#school based service | x x**

**yes#community bed | x x**

**missing observations#emergency service | x x**

**|**

**final_emotionalneglect#final_ipv |**

**no#yes | x x**

**|**

**final_emotionalneglect#final_famsubsuse |**

**missing observations#yes | x x**

**|**

**final_emotionalneglect#final_fammentalillness |**

**no#missing observations | x x**

**|**

**final_emotionalneglect#final_famprison |**

**no#missing observations | x**

**|**

**final_physicalneglect#final_diagnosis |**

**yes#neurodevelopmental | x**

**yes#obsessive-compulsive | x x**

**missing observations# depressive | x x**

**missing observations#schizophrenia/psychotic | x x**

**|**

**final_physicalneglect#final_programtype |**

**no#school based service | x x**

**no#specialized service | x x**

**yes#specialized service | x x**

**|**

**final_physicalneglect#final_famsubsuse |**

**yes#no | x x**

**yes#missing observations | x x**

**|**

**final_parentsdivorced#final_residence |**

**yes#urban/metro/mod metro inf | x**

**|**

**final_parentsdivorced#final_diagnosis |**

**no#disruptive/impulse-control/conduct | x x**

**yes#schizophrenia/psychotic | x x**

**|**

**final_parentsdivorced#final_programtype |**

**no#school based service | x x**

**|**

**final_parentsdivorced#final_famsubsuse |**

**missing observations#missing observations | x x**

**|**

**final_parentsdivorced#final_fammentalillness |**

**no#missing observations | x x**

**|**

**final_ipv#final_diagnosis |**

**no#bipolar | x**

**yes#personality | x x**

**|**

**final_ipv#final_programtype |**

**no#day treatment | x x**

**missing observations#specialized service | x x**

**|**

**final_famsubsuse#final_diagnosis |**

**no#gender dysphoria | x x**

**yes#gender dysphoria | x x**

**yes#neurodevelopmental | x x**

**yes#psychosocial factor | x x**

**|**

**final_famsubsuse#final_fammentalillness |**

**yes#no | x x**

**|**

**final_famsubsuse#final_famprison |**

**yes#missing observations | x x**

**missing observations#no | x x**

**|**

**final_fammentalillness#final_diagnosis |**

**no#others | x x**

**yes#obsessive-compulsive | x x**

**missing observations# depressive | x x**

**missing observations#disruptive/impulse-control/conduct | x x**

**|**

**final_fammentalillness#final_programtype |**

**no#specialized service | x x**

**yes#inpatient unit | x x**

**|**

**final_famprison#final_residence |**

**no#rural/mod urban inf/rural centre | x x**

**yes#missing observation | x x**

**|**

**final_famprison#final_diagnosis |**

**yes#psychosocial factor | x x**

**yes#substance related | x x**

**missing observations# depressive | x**

**missing observations#personality | x**

**|**

**final_sex#c.final_age |**

**female | x x**

**|**

**final_residence#c.final_age |**

**urban/metro/mod metro inf | x x**

**|**

**final_sex#final_residence |**

**male#rural/mod urban inf/rural centre | x x**

**|**

**final_diagnosis#final_programtype |**

**anxiety#community bed | x x**

**anxiety#inpatient unit | x x**

**depressive#outpatient service | x x**

**disruptive/impulse-control/conduct#specialized service | x x**

**medical#emergency service | x x**

**medical#inpatient unit | x x**

**neurodevelopmental#emergency service | x x**

**obsessive-compulsive#community clinic | x x**

**psychosocial factor#specialized service | x x**

**schizophrenia/psychotic#outpatient service | x**

**substance related#consultation service | x x**

**substance related#inpatient unit | x x**

**|**

**final_programtype#c.final_age |**

**emergency service | x**

**|**

**final_sex#final_diagnosis |**

**female# depressive | x x**

**|**

**final_sex#final_programtype |**

**male#inpatient unit | x x**

**female#school based service | x x**

**neither#consultation service | x x**

**neither#outpatient service | x x**

**|**

**final_residence#final_diagnosis |**

**rural/mod urban inf/rural centre#trauma/stressor related | x x**

**urban/metro/mod metro inf#somatic symptom | x x**

**|**

**final_residence#final_programtype |**

**rural/mod urban inf/rural centre#consultation service | x x**

**rural/mod urban inf/rural centre#day treatment | x x**

**urban/metro/mod metro inf#consultation service | x x**

**urban/metro/mod metro inf#emergency service | x x**

**urban/metro/mod metro inf#inpatient unit | x x**

**unclassified/unknown#community clinic | x x**

**missing observation#consultation service | x x**

**missing observation#inpatient unit | x x**

**missing observation#outpatient service | x x**

**|**

**_cons | x x**

**-------------------------------------------------------------------------------------**

**Legend:**

**x - estimated**
