# Supplementary material for: Can the adverse childhood experiences (ACEs) checklist be utilized to predict emergency department visits among children and adolescents?
Source: BMC Med Res Methodol. 2021 Sep 25;21:195. doi: 10.1186/s12874-021-01392-w (PMC8465692; doi:10.1186/s12874-021-01392-w)
Supplement: Supplementary file 2 — Additional file 2. Results of subgroup analyses. [file 12874_2021_1392_MOESM2_ESM.docx]

# Can the Adverse Childhood Experiences (ACEs) checklist be utilized to predict emergency department visits among children and adolescents?

Asmita Bhattarai, MPH ^a, b^, Gina Dimitropoulos, PhD ^b, c^, Brian Marriott ^c, d^, Jaime Paget ^d^, Andrew G.M. Bulloch, PhD ^a, b, e^, Suzanne C. Tough ^a, f^, Scott B. Patten, MD, PhD ^a, b, e^

^a^ Department of Community Health Sciences, Cumming School of Medicine, University of Calgary, 3280 Hospital Drive NW, Calgary, AB, Canada. T2N4Z6.

^b^ Mathison Centre for Research & Education, University of Calgary, 3280 Hospital Drive NW, Calgary, AB, Canada. T2N4Z6.

^c^ Faculty of Social Work, University of Calgary, 2500 University Dr NW, Calgary, AB, Canada. T2N 1N4

^d^ Addiction and Mental Health, Alberta Health Services- Calgary Zone, Canada

^e^ Department of Psychiatry, Cumming School of Medicine, University of Calgary, 2500 University Dr NW, Calgary, AB, Canada. T2N 1N4.

^f^ Department of Pediatrics, Cumming School of Medicine, University of Calgary, 2500 University Dr NW, Calgary, AB, Canada. T2N 1N4.

# Corresponding Author:

Asmita Bhattarai, PhD Candidate (Epidemiology)

Department of Community Health Sciences, Cumming School of Medicine, University of Calgary, 3280 Hospital Drive NW, Calgary, AB, Canada. T2N4Z6.

(T) (587) 917-5060

(E) [asmita.bhattarai1@ucalgary.ca](mailto:asmita.bhattarai1@ucalgary.ca) (ORCID) 0000-0001-6689-7218

Additional file 2: Results of subgroup analyses

# Supplementary Table 1: Discrimination performance of LASSO logistic regression models in the validation dataset predicting mental illness related ED visits among children and adolescents

| Model | Training data | Validation data | | | | | |
| --- | --- | --- | --- | --- | --- | --- | --- |
|  | AUC | AUC | Sensitivity% | Specificity% | PPV% | NPV% | Overall % |
| Cross Validated | 0.741 | 0.726 | 1.4 | 99.5 | 40.0 | 85.2 | 84.9 |
| Adaptive | 0.748 | 0.712 | 6.1 | 97.5 | 29.8 | 85.5 | 83.8 |

# Supplementary Table 2: Calibration matrix of the LASSO regression models in the validation dataset predicting mental illness related ED visits among children and adolescents

| Model | Predicted probability of ED visit % | Observed ED visit % (95%CI) | Risk stratification capacity  N (%) | Out of sample deviance ratio |
| --- | --- | --- | --- | --- |
| Cross Validated | 0-24 | 11.2 (9.5,13.1) | 1204 (79.0) | 0.082 |
|  | 25-49 | 28.6 (23.9,33.9) | 311 (20.4) |  |
|  | 50-74 | 40.0 (13.4,74.2) | 10 (0.7) |  |
|  | 75-100 | No observations | 0 (0) |  |
| Adaptive | 0-24 | 11.0 (9.3,13.0) | 1142 (74.9) | 0.060 |
|  | 25-49 | 26.2 (21.8,31.2) | 336 (22.0) |  |
|  | 50-74 | 27.8 (15.3,45.0) | 36 (2.4) |  |
|  | 75-100 | 36.4 (12.4,69.8) | 11 (0.7) |  |

# Supplementary Table 3: Discrimination performance of the LASSO regression models in the validation dataset among adolescents (10 years and above)

| Model | Training data | Validation data | | | | | |
| --- | --- | --- | --- | --- | --- | --- | --- |
|  | AUC | AUC | Sensitivity% | Specificity% | PPV% | NPV% | Overall% |
| Cross Validated | 0.658 | 0.587 | 33.7 | 78.4 | 53.6 | 61.5 | 59.4 |
| Adaptive | 0.673 | 0.586 | 42.3 | 68.4 | 49.8 | 61.6 | 57.3 |

# Supplementary Table 4: Calibration matrix of the LASSO regression models in the validation dataset among adolescents (10 years and above)

| Model | Predicted probability of ED visit % | Observed ED visit % (95%CI) | Risk stratification capacity  N (%) | Out of sample deviance ratio |
| --- | --- | --- | --- | --- |
| Cross Validated | 0-24 | 28.6 (14.5, 48.6) | 28 (2.4) | 0.012 |
|  | 25-49 | 38.8 (35.5, 42.2) | 819 (70.8) |  |
|  | 50-74 | 53.5 (48.0, 59.1) | 310 (26.8) |  |
|  | 75-100 | No observations | 0 (0) |  |
| Adaptive | 0-24 | 27.2 (19.0, 37.3) | 92 (8.0) | -0.041 |
|  | 25-49 | 40.0 (36.3, 43.9) | 647 (55.9) |  |
|  | 50-74 | 49.9 (44.9, 54.9) | 381 (32.9) |  |
|  | 75-100 | 48.7 (32.7, 64.9) | 37 (3.2) |  |
