# Supplementary material for: Can the adverse childhood experiences (ACEs) checklist be utilized to predict emergency department visits among children and adolescents?
Source: BMC Med Res Methodol. 2021 Sep 25;21:195. doi: 10.1186/s12874-021-01392-w (PMC8465692; doi:10.1186/s12874-021-01392-w)
Supplement: Supplementary file 3 — Additional file 3. Results of sensitivity analyses. [file 12874_2021_1392_MOESM3_ESM.docx]

# Can the Adverse Childhood Experiences (ACEs) checklist be utilized to predict emergency department visits among children and adolescents?

Asmita Bhattarai, MPH ^a, b^, Gina Dimitropoulos, PhD ^b, c^, Brian Marriott, MSc ^c, d^, Jaime Paget, BA ^d^, Andrew G.M. Bulloch, PhD ^a, b, e^, Suzanne C. Tough ^a, f^, Scott B. Patten, MD, PhD ^a, b, e^

^a^ Department of Community Health Sciences, Cumming School of Medicine, University of Calgary, 3280 Hospital Drive NW, Calgary, AB, Canada. T2N4Z6.

^b^ Mathison Centre for Research & Education, University of Calgary, 3280 Hospital Drive NW, Calgary, AB, Canada. T2N4Z6.

^c^ Faculty of Social Work, University of Calgary, 2500 University Dr NW, Calgary, AB, Canada. T2N 1N4

^d^ Addiction and Mental Health, Alberta Health Services- Calgary Zone, Canada

^e^ Department of Psychiatry, Cumming School of Medicine, University of Calgary, 2500 University Dr NW, Calgary, AB, Canada. T2N 1N4.

^f^ Department of Pediatrics, Cumming School of Medicine, University of Calgary, 2500 University Dr NW, Calgary, AB, Canada. T2N 1N4.

# Corresponding Author:

Asmita Bhattarai, PhD Candidate (Epidemiology)

Department of Community Health Sciences, Cumming School of Medicine, University of Calgary, 3280 Hospital Drive NW, Calgary, AB, Canada. T2N4Z6.

(T) (587) 917-5060

(E) [asmita.bhattarai1@ucalgary.ca](mailto:asmita.bhattarai1@ucalgary.ca) (ORCID) 0000-0001-6689-7218

Additional file 3: Results of sensitivity analyses

# Supplementary Table 1: Discrimination performance of LASSO logistic regression models in the validation dataset (predicting all-cause ED visits using ACE scores)

| Model | Training data | Validation data | | | | | |
| --- | --- | --- | --- | --- | --- | --- | --- |
|  | AUC | AUC | Sensitivity% | Specificity% | PPV% | NPV% | Overall % |
| Cross Validated | 0.659 | 0.618 | 33.2 | 81.9 | 55.3 | 64.5 | 62.3 |
| Adaptive | 0.667 | 0.621 | 38.9 | 76.8 | 53.1 | 65.1 | 61.6 |

# Supplementary Table 2: Calibration matrix of the LASSO regression models in the validation dataset (predicting all-cause ED visits using ACE scores)

| Model | Predicted probability of ED visit % | Observed ED visit % (95%CI) | Out of sample deviance ratio |
| --- | --- | --- | --- |
| Cross Validated | 0-24 | 18.8 (9.9,32.7) | 0.030 |
|  | 25-49 | 36.2 (33.4,39.1) |  |
|  | 50-74 | 55.3 (50.2,60.3) |  |
|  | 75-100 | - |  |
| Adaptive | 0-24 | 20.8 (15.0,28.1) | 0.013 |
|  | 25-49 | 37.1 (34.1,40.3) |  |
|  | 50-74 | 53.4 (48.6,58.1) |  |
|  | 75-100 | 48.0 (28.8,67.8) |  |

# Supplementary Table 3: Discrimination performance of the LASSO regression models in the validation dataset (predicting all-cause ED visits using threat-based childhood adversities)

| Model | Training data | Validation data | | | | | |
| --- | --- | --- | --- | --- | --- | --- | --- |
|  | AUC | AUC | Sensitivity% | Specificity% | PPV% | NPV% | Overall% |
| Cross Validated | 0.657 | 0.607 | 27.0 | 82.8 | 51.4 | 62.7 | 60.3 |
| Adaptive | 0.666 | 0.608 | 38.1 | 75.0 | 50.7 | 64.3 | 60.1 |

# Supplementary Table 4: Calibration matrix of the LASSO regression models in the validation dataset (predicting all-cause ED visits using threat-based childhood adversities)

| Model | Predicted probability of ED visit % | Observed ED visit % (95%CI) | Out of sample deviance ratio |
| --- | --- | --- | --- |
| Cross Validated | 0-24 | 19.5 (9.9,35.0) | 0.024 |
|  | 25-49 | 37.9 (35.1,40.7) |  |
|  | 50-74 | 51.4 (45.9,56.8) |  |
|  | 75-100 | - |  |
| Adaptive | 0-24 | 21.0 (15.6,27.7) | 0.004 |
|  | 25-49 | 38.7 (35.5,41.9) |  |
|  | 50-74 | 50.9 (46.2,55.6) |  |
|  | 75-100 | 45.8 (26.6,66.4) |  |

# Supplementary Table 5: Discrimination performance of the LASSO regression models in the validation dataset (predicting all-cause ED visits using deprivation-based childhood adversities)

| Model | Training data | Validation data | | | | | |
| --- | --- | --- | --- | --- | --- | --- | --- |
|  | AUC | AUC | Sensitivity% | Specificity% | PPV% | NPV% | Overall% |
| Cross Validated | 0.681 | 0.610 | 33.4 | 79.6 | 52.4 | 63.9 | 61.0 |
| Adaptive | 0.693 | 0.607 | 38.6 | 73.8 | 49.8 | 64.1 | 59.6 |

# Supplementary Table 6: Calibration matrix of the LASSO regression models in the validation dataset (predicting all-cause ED visits using deprivation-based childhood adversities)

| Model | Predicted probability of ED visit % | Observed ED visit % (95%CI) | Out of sample deviance ratio |
| --- | --- | --- | --- |
| Cross Validated | 0-24 | 20.7 (14.1,29.4) | 0.022 |
|  | 25-49 | 37.7 (34.8,40.7) |  |
|  | 50-74 | 52.9 (47.8,57.9) |  |
|  | 75-100 | 33.3 (8.9,71.9) |  |
| Adaptive | 0-24 | 24.4 919.1,30.7) | -0.029 |
|  | 25-49 | 38.9 (35.6,42.2) |  |
|  | 50-74 | 49.5 944.8,54.3) |  |
|  | 75-100 | 51.9 (38.4,65.0) |  |
